# Improvement in the active management of the third stage of labor for the prevention of postpartum hemorrhage in Tanzania: a cross-sectional study

**DOI:** 10.1186/s12884-018-1873-3

**Published:** 2018-06-13

**Authors:** Dunstan R. Bishanga, John Charles, Gaudiosa Tibaijuka, Rita Mutayoba, Mary Drake, Young-Mi Kim, Marya Plotkin, Neema Rusibamayila, Barbara Rawlins

**Affiliations:** 1 Jhpiego Tanzania, Box 9170, Dar es Salaam, PO Tanzania; 2 Jhpiego Baltimore, Baltimore, MD USA; 3 Department of Health Sciences, Global Health, University of Groningen/University Medical Center Groningen, Groningen, The Netherlands; 4 Tanzania Ministry of Health, Community Development, Gender, Elderly and Children, Dar es Salaam, Tanzania; 5 PACT TANZANIA, Box 6348, Dar es Salaam, PO Tanzania; 6 Amref Health Africa, Box 2773, Dar es Salaam, PO Tanzania

**Keywords:** Postpartum hemorrhage, Quality of care, Active management of the third stage of labor, AMTSL, Uterotonic, Obstetric complications, Tanzania

## Abstract

**Background:**

Tanzania has a maternal mortality ratio of 556 per 100,000 live births, representing 21% of all deaths of women of reproductive age. Hemorrhage, mostly postpartum hemorrhage (PPH), is estimated to cause at least 25% of maternal deaths in Tanzania. In 2008, the Ministry of Health, Community Development, Gender, Elderly and Children launched interventions to improve efforts to prevent PPH. Competency-based training for skilled birth attendants and ongoing quality improvement prioritized the practice of active management of the third stage of labor (AMTSL).

**Methods:**

A cross-sectional study was conducted in 52 health facilities in Tanzania utilizing direct observations of women during labor and delivery. Observations were conducted in 2010 and, after competency-based training and quality improvement interventions in the facilities, in 2012. A total of 489 deliveries were observed in 2010 and 558 in 2012. Steps for AMTSL were assessed using a standardized structured observation checklist that was based on World Health Organization guidelines.

**Results:**

The proportion of deliveries receiving all three AMTSL steps improved significantly by 19 percentage points (*p* < 0.001) following the intervention, with the most dramatic increase occurring in health centers and dispensaries (47.2 percentage point change) compared to hospitals (5.2 percentage point change). Use of oxytocin for PPH prevention rose by 37.1 percentage points in health centers and dispensaries but remained largely the same in hospitals, where the baseline was higher. There was substantial improvement in the timely provision of uterotonics (within 3 min of birth) across all facilities (*p* = 0.003). Availability of oxytocin, which was lower in health centers and dispensaries than hospitals at baseline, rose from 73 to 94% of all facilities.

**Conclusion:**

The quality of PPH prevention increased substantially in facilities that implemented competency-based training and quality improvement interventions, with the most dramatic improvement seen at lower-level facilities. As Tanzania continues with efforts to increase facility births, it is imperative that the quality of care also be improved by promoting use of up-to-date guidelines and ensuring regular training and mentoring for health care providers so that they adhere to the guidelines for care of women during labor. These measures can reduce maternal and newborn mortality.

**Electronic supplementary material:**

The online version of this article (10.1186/s12884-018-1873-3) contains supplementary material, which is available to authorized users.

## Background

Globally, maternal mortality is a major public health problem, with an estimated 303,000 deaths occurring in 2015. Sub-Saharan Africa accounted for 195,000—or almost two-thirds—of those deaths [1]. Tanzania is a substantial contributor to maternal mortality, with a maternal mortality ratio of 556 per 100,000 live births during the 10-year period before the 2015/2016 Demographic and Health survey. Maternal mortality accounts for 21% of deaths of women of reproductive age [2]. Maternal mortality in Tanzania has not significantly changed over the last decade despite a positive trend in institutional deliveries, which rose from 47% in 2004 to over 60% in 2015 [2–4]. This gap suggests the urgent need to improve quality of care in health facilities to reduce maternal and perinatal morbidity and mortality, particularly around labor and delivery and the immediate postnatal period [5].

Studies have shown that improving the quality of care can address obstetric complications and reduce preventable perinatal and maternal deaths [6–9]. The World Health Organization (WHO) defines quality of care as “the extent to which health care services provided to individuals and patient populations improve desired health outcomes. In order to achieve this, health care must be safe, effective, timely, efficient, equitable and people-centered” [8]. WHO supports the provision of high-quality intrapartum care to save lives through use of up-to-date guidelines and standards. Evidence-based practices for routine care and management of complications form part of the eight domains in the WHO quality-of-care framework for maternal and newborn health [5].

Obstetric hemorrhage remains the leading cause of maternal mortality in low-income countries; it accounts for up to 34% of maternal deaths in Africa [10] and at least one-fourth of maternal deaths in Tanzania [11, 12]. Despite the fact that it is largely preventable, postpartum hemorrhage (PPH) is the most common and most deadly form of obstetric bleeding [13]. One assessment found that PPH was the second leading cause of maternal deaths at Muhimbili National Hospital in Dar es Salaam [14]. The main PPH prevention measure recommended in low- and middle-income countries (LMICs) is active management of the third stage of labor (AMTSL), which WHO recommends for all deliveries, in LMIC settings [15]. As defined in 2003 by the International Confederation of Midwives (ICM) and International Federation of Gynecology and Obstetrics (FIGO), AMTSL had three main components: (1) administration of a uterotonic within 1 min of birth (the “relaxed” definition is within 3 min of birth); (2) delivery of the placenta by controlled cord traction (CCT); and (3) uterine massage [16]. In 2012, WHO revised the definition to emphasize provision of uterotonic (preferably oxytocin) in the third stage of labor [15, 17]; CCT and sustained uterine massage are no longer recommended for uncomplicated deliveries. Continued monitoring of the uterus for 2 h after birth is recommended, with fundal massage if the uterus is soft [17].

There is a dearth of published information on the observed quality of delivery care in low-income countries, and few studies include observations of actual PPH prevention care. A study in seven countries (Benin, Ethiopia, Tanzania, Indonesia, El Salvador, Honduras, and Nicaragua) conducted by Stanton et al. in 2005–2006 used direct observation to assess AMTSL and found correct use in 1.7 to 39.6% of deliveries in each country, with Tanzania being among the lowest; this analysis used the ICM-FIGO definition of AMTSL, with timing relaxed to 3 min [18]. In 2009, Mfinanga et al. re-analyzed the Tanzanian data from this study to account for data collection problems and changing definitions of ergometrine dosages. They found that correct practice of AMTSL was 17%, using the relaxed definition and recommended uterotonic at the time (i.e., ergometrine within 3 min of delivery) [19]. More recently, Bartlett et al. reported substantial variations in the use of AMTSL within 3 min of birth in six sub-Saharan countries; direct observation of facility-based AMTSL found that correct use ranged from 28 to 62% across these countries and was 42% for Tanzania. The Tanzania finding draws on the same 2010 data as the current study but includes Zanzibar; the analysis presented in this paper is limited to the Tanzania mainland [20].

In 2008 the Ministry of Health, Community Development, Gender, Elderly and Children (MoHCDGEC) (formerly the Ministry of Health and Social Welfare) adopted routine use of AMTSL in all deliveries for PPH prevention. The protocol recommends oxytocin as the preferred uterotonic, with ergometrine and misoprostol recommended where oxytocin is not available [11]. Routine provision of AMTSL was subsequently scaled up by the ministry and implementing partners, including the Mothers and Infants Safe, Healthy, Alive (MAISHA) program, a 5-year program funded by the United States Agency for International Development (USAID) and led by Jhpiego. The MAISHA program sought to improve the quality of maternal and newborn care by providing competency-based training for health care providers and a quality improvement intervention for maternal and newborn health services.

The MAISHA program, with support from the Maternal and Child Health Integrated Program, conducted a cross-sectional study using direct observations of deliveries to assess the quality of key elements of maternal and newborn care in selected health facilities in 12 regions of Tanzania and to evaluate the intervention. Observations were conducted in 2010 and then repeated in 2012 after competency-based training and quality improvement interventions were implemented in the facilities.

This paper compares the two rounds of observations to provide an in-depth assessment of the quality of PPH prevention services in Tanzania before and after a quality improvement program. Thus, it not only provides critical insight into the state of PPH preventative services but also contributes to evidence on the potential impact of quality improvement programs on specific areas of intrapartum care. The study results will be useful for policy makers, program planners, and key stakeholders in improving the quality of intrapartum care in the LMIC setting.

## Methods

### Study design

A cross-sectional health facility assessment using direct observations of the quality of care during labor and delivery was conducted in 12 of Tanzania’s 31 regions (Tanga, Mtwara, Lindi, Arusha, Kilimanjaro, Morogoro, Manyara, Tabora, Pwani, Kigoma, Ruvuma and Iringa) in 2010 and 2012. These included all Phase 1 regions of the MAISHA program, which was eventually rolled out to the rest of the country. Quality improvement interventions were introduced shortly before the 2010 study, which was intended to be a baseline assessment. The 2012 assessment was designed to examine the effects of the MAISHA program interventions. The 2010 assessment included 52 facilities: 12 hospitals and 40 health centers and dispensaries. Two lower-level facilities were dropped from the 2012 assessment because they did not receive interventions as planned due to staffing challenges.

### Interventions to improve quality of PPH services in MAISHA program sites

The MAISHA program provided technical assistance to the MoHCDGEC to develop and disseminate standardized maternal and newborn health care guidelines based on WHO recommendations, including for PPH prevention. The program developed clinical standards and training materials, including PPH prevention job aids. The program also provided support at central and local levels to roll out the guidelines and learning resources. Results from the baseline assessment were disseminated to key stakeholders and joint action plans were developed locally with facility and district teams. The plans were designed to address gaps identified in the provision of high-quality care.

Capacity building was a core component of the program, which sought to equip service providers, supervisors and managers with knowledge, attitudes and skills to enable provision of quality maternal care according to established standards. The program facilitated competency-based training, coaching and mentoring to strengthen the provision of routine maternal and newborn care, including the administration of uterotonics as part of AMTSL and emergency obstetric care (EmOC) signal functions that address management of PPH. Providers in target facilities received supervision and coaching on a quarterly basis, with gaps in skills addressed on site by trained supervisors. The mean length of the MAISHA program in these facilities was 25 months, with a range of 17 to 41 months.

**Fig. 1:**
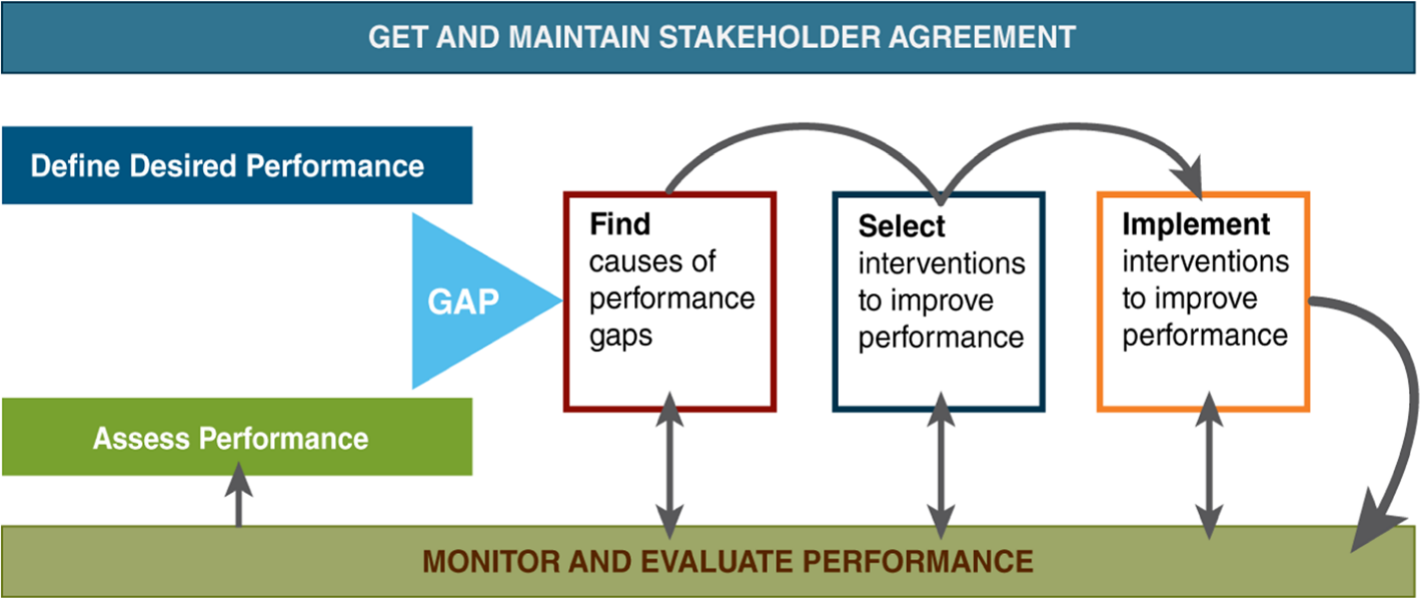
Cycle of performance improvement

Under the MAISHA program, facilities formed quality improvement teams (QITs), which received training and ongoing supportive supervision and mentorship. QITs included representatives from different departments within the facility and used the Standards-Based Management and Recognition approach, which utilizes a “Plan-Do-Study-Act” cycle to improve the quality of maternal and newborn care [21]. Subsequently, the QITs facilitated quality circles, referred to as work improvement teams (WITs), which used a participatory management technique that enlists the help of health care providers in solving problems related to the provision of care. WITs included staff providing care in labor and delivery wards, antenatal and postnatal wards and antenatal clinics. Both teams met regularly to assess actual performance against desired performance, identify performance gaps, select solutions to improve performance and prepare action plans to implement the performance-improvement solutions (Fig. 1).

Trained external assessors from the MAISHA program and MoHCDGEC performed facility assessments annually or upon request by facility staff/management. Facilities that achieved an 80% score on maternal and newborn clinical performance standards were formally recognized by the MoHCDGEC.

The program trained and provided quality improvement support to 1593 providers and supervisors. Additionally, key health managers at regional, district and facility levels were oriented on the quality improvement tools so they could support the WITs. The program trained 921 service providers in EmOC, including AMTSL, in the 52 facilities. Providers of maternity services who did not receive training initially were reached through onsite supervision and mentorship.

### Sampling

The sampling of facilities was done purposively and included regional hospitals, health centers and dispensaries. Inclusion criteria for health centers and dispensaries included conducting at least one delivery per day. Two health centers were subsequently dropped from the MAISHA program due to inadequate staffing, so that only 50 facilities were reassessed in 2012.

This study was designed to capture change in AMTSL practice by estimating the number of deliveries that needed to be observed to see a change of up to 50% in the provision of AMTSL. We used the Prevention of Postpartum Hemorrhage Initiative national survey (POPPHI-2006) as the basis for the proportion of women who correctly received AMTSL, using the FIGO/ICM definition with a uterotonic administered within 1 min of delivery. This value was 7% [22]. Assuming a change of up to 50% in the practice, with a desired power of 80% and precision of 95%, the sample needed to include observations of 449 deliveries.

### Data collection tools and procedure

Data collectors used standardized tools, including the Service Provision Assessment’s provider interview guide and WHO’s clinical guidelines [23]. The routine labor and delivery clinical observation checklist was adapted from an instrument used by Stanton et al. [18].

A daily quota of deliveries to be observed during the 2- to 4-day data collection period was calculated per facility, based upon the expected number of deliveries per day at that facility. Women admitted for an emergency caesarean section were not observed, nor were those who needed caesarean section after the start of labor.

The following procedures were used during data collection:

1. **Observations of labor and delivery**: In each health facility, trained data collectors used a structured observation checklist to observe and document care provided to women giving birth (Additional file 1). Data collectors observed any provider working at the time of the assessment, regardless of whether or not they had received training under the MAISHA program. Data collectors observed key steps in the provision of care to women with uncomplicated deliveries. The first stage of labor involved intermittent observation, while the second and third stages of labor involved continuous observation until one hour after birth. Performance or non-performance of each component of AMTSL was documented, including the type of uterotonic used.
2. **Facility audit/inventory for presence of uterotonic drugs** (Additional file 2): A standardized audit was conducted to assess selected components of facility readiness; the audit included both the maternity ward and facility pharmacy (drug storage rooms). Data collectors visually confirmed the presence of at least one dose of oxytocin, ergometrine and/or misoprostol in the maternity ward and checked the drug expiry date; expired drugs were excluded from the inventory. Any field missing a value was assumed to indicate the absence of the drug.

Data collectors were selected from a pool of national basic emergency obstetric and newborn care trainers. They underwent a two-week training on study methods, research ethics and observation practices using the study tools and tablets. Inter-rater reliability was assessed during training and repeated until the observers had a high level of agreement. The training also included clinical updates on intrapartum and immediate postpartum care and 2 days of practice using the tools in two non-study health facilities. To reduce bias, data collectors were assigned to study sites where they had never worked or conducted training. Fourteen of the 20 data collectors employed in 2012 had also participated in the 2010 data collection.

Data collectors entered observations and other data into tablets (Samsung Galaxy with Mobile Data Studio software) that were pre-populated with the data collection forms. The observers could observe multiple deliveries at the same time by flipping between files. No more than three deliveries were observed at any given time.

### Data management

The data entry applications on the tablet controlled question flow, data skips along with range, consistency and data quality checks. Every evening, supervisors would transfer data to the server. Upon receipt, further quality checks were performed before porting the cleaned data over to an SQL Server database with a password-protected web portal for analyzing and displaying the data.

### Analysis

Variables were created based on “yes, observed”/ “no, not observed” responses to observational checklist items, with “don’t know” responses excluded. Stata Statistical Software 12.0 was used to analyze data to generate descriptive statistics, including means and frequencies. Cross-tabulations using chi-square tests of significance were used to compare quality-of-care indicators between 2010 and 2012. Post-stratification weights were applied to labor and delivery observations to account for differences between the numbers of observed and expected cases based on health management information system records of health facility caseloads of deliveries. A *p* value of 0.05 was considered statistically significant in all analyses.

The AMTSL components’ analysis was based on the FIGO/ICM AMTSL definition, which was widely used in 2008 at the conception of the study. We analyzed administration within 1 min of birth (per the FIGO/ICM definition) and within 3 min (per the “relaxed” definition). Analyses of timing of uterotonic administration were based on observers’ recording of the exact times. Missing/invalid values were assumed to not adhere to either the 1-min or 3-min guidelines.

## Results

### Sample of observed deliveries

A total of 489 deliveries were observed at 52 health facilities (12 hospitals and 40 lower-level health facilities) in 2010, and 558 deliveries were observed at 50 of these same facilities (12 hospitals and 38 lower-level facilities) in 2012 (Table 1). In 2010, of the 415 women who were observed during the third stage of labor, 403 received any uterotonic (97.1%); in 2012, of the 502 women who were observed during the third stage of labor, 451 received any uterotonic (89.8%). The proportion of women receiving any uterotonic regardless of timing fell by 7.3 percentage points between 2010 and 2012, which was not statistically significant (data not shown).

**Table 1:** Description of deliveries observed at health facilities in 2010 and 2012

Description	Hospitals		Health centers & dispensaries		All health facilities	
	2010 (*n* = 12)	2012 (*n* = 12)	2010 (*n* = 40)	2012 (*n* = 38)	2010 (*n* = 52)	2012 (*n* = 50)
Total number of deliveries observed	195	344	294	214	489	558
Number of deliveries with third stage of labor observed	164	311	251	191	415	502
Number of deliveries with third stage of labor observed and given any uterotonic	164	283	239	168	403	451

*n* number of health facilities

### Frequency and quality of provision of AMTSL

**Fig. 2:**
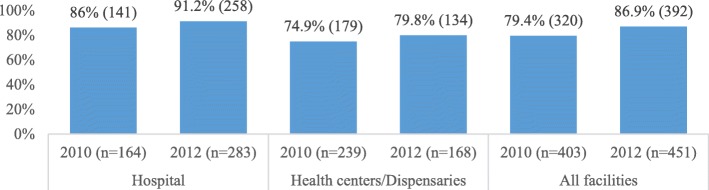
Among women who received any uterotonic, proportion receiving it within 3 min of childbirth

The provision of all three steps of AMTSL (uterotonic, CCT, and uterine massage) increased across all health facilities by 19 percentage points from 2010 to 2012 (*p* < 0.001), largely due to increases in uterine massage (28.8 points) and CCT (15.5 points); there was little change in uterotonic use (Table 2). However, a further analysis limited only to those women who received a uterotonic reveals a significant improvement in the timing of its administration: the proportion of women who received the uterotonic on schedule (i.e., within 3 min after birth) increased by 7.5 percentage points across all facilities (*p* = 0.003) (Fig. 2).

**Table 2:** Observed provision of AMTSL by level of health facility

AMTSL steps	Hospitals n (%)			Health centers & dispensaries n (%)				All health facilities n (%)				
	2010 (*N* = 164)	2012 (*N* = 311)	% point change	*p* value	2010 (*N* = 251)	2012 (*N* = 191)	% point change	*p* value	2010 (*N* = 415)	2012 (*N* = 502)	% point change	*p* value
All steps	80 (48.8)	168 (54.0)	5.2	0.301	55 (21.9)	132 (69.1)	47.2	< 0.001	170 (41.0)	301 (60.0)	19.0	< 0.001
Provision of any uterotonic within 3 min	141 (86.0)	258 (83.0)	−3.0	0.396	179 (71.3)	134 (70.2)	−1.1	0.801	320 (77.1)	392 (78.1)	1.0	0.718
Controlled cord traction	121 (73.8)	289 (92.9)	19.1	< 0.001	185 (73.7)	159 (83.2)	9.5	0.017	306 (73.7)	448 (89.2)	15.5	< 0.001
Uterine massage	99 (60.4)	277 (89.1)	28.7	< 0.001	137 (54.6)	153 (80.1)	25.5	< 0.001	236 (56.9)	430 (85.7)	28.8	< 0.001

*AMTSL* active management of third stage of labor; *N* number of deliveries; n = frequency

Lower-level health facilities made greater gains than hospitals in the provision of all steps of AMTSL (47.2 percentage point increase versus 5.2 percentage points, *p* < 0.001). Performance of CCT rose significantly (*p* < 0.001) by 15.5 percentage points across all levels of facilities, with greater gains in hospitals (19.1 percentage points, *p* < 0.001). The performance of uterine massage following delivery of the placenta increased from 56.9 to 85.7% across all facility types (*p* < 0.001) (Table 2).

Substantial gains were made in oxytocin use at lower-level facilities, rising from 58.1 to 95.2% of all uterotonics used (*p* < 0.001). There was virtually no change at hospitals because oxytocin use was already universal in those facilities at baseline (Table 3).

**Table 3:** Type of uterotonic used for AMTSL by level of health facility

Uterotonic type	Hospitals n (%)				Health centers & dispensaries n (%)				All health facilities n (%)			
	2010 (*N* = 164)	2012 (*N* = 283)	% point change	*p* value	2010 (*N* = 239)	2012 (*N* = 168)	% point change	*p* value	2010 (*N* = 403)	2012 (*N* = 451)	% point change	*p* value
Oxytocin	164 (100)	282 (99.7)	−0.3	0.482	139 (58.1)	160 (95.2)	37.1	< 0.001	303 (75.2)	442 (98.0)	22.8	< 0.001
Ergometrine	0 (0)	0 (0)	0	-	74 (31.0)	8 (4.8)	−26.2	< 0.001	74 (18.4)	8 (1.8)	−16.6	< 0.001
Misoprostol	0 (0)	1 (0.4)	0.4	0.27	26 (10.9)	0 (0)	−10.9	< 0.001	26 (6.4)	1 (0.2)	−6.2	< 0.001

*N* number of deliveries; *n* frequency

### Facility readiness to provide AMTSL: Availability of drugs and service delivery guidelines

**Fig. 3:**
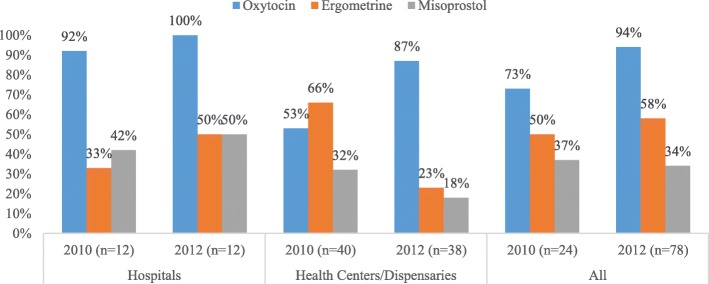
Availability of uterotonics in assessed health facilities on assessment day

Figure 3 shows the availability of each type of uterotonic in the facilities on the day of the study team’s visit. Generally, availability of the first choice uterotonic (oxytocin) increased significantly (*p* < 0.001) across all facility types from 73% of at baseline to 94% at the time of reassessment in 2012. The increase was greater at lower-level facilities (from 53 to 87%) compared with hospitals (from 92 to 100%). In lower-level facilities the availability of the second choice uterotonic, ergometrine, dropped by 43 percentage points (*p* < 0.001) and the availability of misoprostol dropped by 14 percentage points (p < 0.001); an increase in both was observed in hospitals (Fig. 3).

Out of the 52 health facilities surveyed in 2010, only 19 facilities (37%) had guidelines for uncomplicated delivery and 28 (54%) had guidelines for emergency obstetric and newborn care. Following interventions under the MAISHA program, both guidelines became more widely available (p < 0.001): 44 of 50 health facilities surveyed in 2012 (88%) had guidelines for uncomplicated delivery and 46 (92%) had guidelines for emergency obstetric and newborn care (data not shown in the tables).

## Discussion

The study’s findings showed that overall PPH prevention practices improved significantly in hospitals and lower-level health facilities for all three components of AMTSL emphasized by the MAISHA program. The program followed the original definition proposed by ICM and FIGO [17], however, WHO’s current emphasis is on the use of a uterotonic for prevention of PPH during the third stage of labor [15, 17]. Although WHO recommendations do not specify the timing, providing a uterotonic within 3 min of birth has been defined as a correct AMTSL practice [18, 20, 23]. Our findings show a small but significant improvement in timing for uterotonic provision, although overall uterotonic use did not change significantly. Any follow-up intervention should consider the latest WHO recommendations, which also include delayed cord clamping.

Health centers and dispensaries registered a significant increase in use of oxytocin for PPH prevention compared to other uterotonics. This was a desired change since national guidelines recommend oxytocin as the drug of choice for PPH. The study also documented significant increases in the availability of oxytocin in health centers and dispensaries as well as in its use for AMTSL. These results are in accord with a study in India that found the availability of oxytocin was associated with administration of the drug for PPH prevention [24].

An observational study conducted in Tanzania in 2005—a few years before national PPH prevention and management guidelines came into effect—concluded that correct AMTSL practice, using the relaxed definition, was as low as 17% [19]. Much has changed since then, including the adoption of oxytocin instead of ergometrine in national policy and the establishment of MoHCDGEC guidelines in 2008. These changes are reflected in the 48.4% baseline measure of correct AMTSL practice prior to the MAISHA intervention in 2010. The current study thus documents how a quality improvement initiative can build on existing progress to further strengthen the practice of AMTSL.

After the implementation of MAISHA program intervention, the proportion of deliveries in which all AMTSL steps were performed correctly rose from 41 to 60%. While this study was not designed to attribute causality to the intervention, the findings strongly suggest that MAISHA program activities yielded these improvements. This effect was especially pronounced at lower-level health facilities, which had a greater need for improvement.

Lower-level facilities in Tanzania have fewer health care providers than hospitals, but they have lower staff turnover. Turnover of trained staff has been reported to adversely affect the quality of services provided in maternal care [25]. This may help explain why lower-level facilities showed greater improvement than hospitals since data collectors observed any provider conducting deliveries, not just those who had received training from the MAISHA program. Thus, the results at lower-level facilities may reflect a more concentrated group of individuals exposed to MAISHA’s quality improvement interventions.

Facility readiness to provide AMTSL, as measured by the availability of guidelines and uterotonics, also improved between the two assessments. Availability of guidelines for uncomplicated delivery, which includes national recommendations on AMTSL for prevention of PPH, increased significantly from baseline to the 2012 assessment. Additionally, the availability of uterotonics (particularly oxytocin) improved, with a significant increase observed in health centers and dispensaries. This may be due to the emphasis on oxytocin as the first choice uterotonic for PPH prevention and management, in line with Tanzania’s guidelines [11]. The observed changes at lower-level facilities is also likely to have resulted from dissemination of PPH prevention and management policy guidelines to the facility level, improved provider knowledge on timely ordering and stock management, as well as increased availability of uterotonics in the labor room.

At the time the MAISHA program was being implemented, the MoHCDGEC was implementing another program that ensured that oxytocin was available for free at national medical stores. This may have enabled health facilities to obtain sufficient oxytocin to meet their needs even with constrained budgets. The improved availability of uterotonics at study sites coincided with interventions supported by the MAISHA program emphasizing timely ordering of supplies by health facilities, which bridged the gap between national policy and practice. This was an important intervention since studies have shown that inconsistent availability of uterotonics could negatively impact the benefits of PPH prevention efforts [24]. Findings from a multi-country survey conducted in 2012 by Smith et al. indicated that the supply of oxytocin was problematic and, in some countries, clients had to pay for oxytocin out of pocket despite national policies to provide it to clients at no cost [25].

An improvement in the availability of oxytocin—the recommended first-line drug of choice—was observed in both hospitals and lower-level health facilities. However, availability increased more markedly in health centers and dispensaries, where coverage was very low at baseline. In surveyed hospitals, availability of oxytocin was high at baseline and universal during the 2012 reassessment. Increased availability of oxytocin should improve the quality of care women receive when giving birth, as was reported in a recent study in western Tanzania where poor availability of essential commodities, such as uterotonics, was one of the bottlenecks for improving quality of care at birth [26].

Higher uterotonic coverage is expected at hospitals than lower-level facilities because hospitals have a stronger supply chain system and more qualified staff. A survey of maternal health experts in Tanzania reported that use of uterotonics immediately following birth was almost universal in hospitals (at 99%), whereas use in lower-level facilities was estimated at less than three-quarters of all births [27], confirming the results of this study.

Despite the widespread availability of oxytocin, some women still did not receive a uterotonic within 3 min of birth. Incorrect timing of administration of oxytocin for AMTSL has been reported as one of the factors affecting AMTSL practice in Tanzania [19]. A recent study of variations in care in Tanzania reported an association between health workforce density and care at birth [26]; in a setting with limited staff undertaking multiple tasks, staff may not have enough time to follow the protocol. This could be one of the contributing factors that prevented some women from receiving appropriate uterotonic provision.

One of the study’s strengths was use of direct observation to assess provision of care. Direct observation overcomes self-report biases and gaps inherent in written documentation. The study team does recognize that with direct observations, the observer can have an effect on the behavior of the person being observed. To strengthen the study and reduce bias, inter-rater reliability measures were applied during standardization of clinical observers.

Because this study was intended to serve as an evaluation of the MAISHA program, the study was not designed to be nationally representative and figures on AMTSL provision should not be interpreted in this way. Another potential limitation is that we did not collect data on, or factor in the presence of, other concurrent interventions that may have affected quality of intrapartum care at study facilities. To our knowledge, however, the MAISHA program was the only ministry-led national initiative that supported capacity building in emergency obstetric and newborn care at the target facilities involved in the study. If there were other initiatives, they must have been ad hoc and very small scale, making them unlikely to account for the overall improvement observed in this study.

In retrospect, the study could have utilized Mfinanga’s estimation of prevalence of AMTSL using the relaxed definition of AMSTL (17%) rather than estimation of prevalence of AMTSL within one minute (7%) from the same study [19]. Despite this, our estimation of AMTSL, based on our sample, was sufficient to achieve statistical significance. Finally, as a cross-sectional study, the data collected on availability of uterotonics did not reflect the history of stock-outs in each facility but rather provided an indication of stock status at a particular moment in time. We believe that despite the limitations, the findings provide considerable insight into health care provider practice around PPH prevention in Tanzania.

Further research is recommended to investigate the wider outcomes and impacts of improvements in AMTSL practices and the availability of oxytocin. A follow-up study at the same study facilities could investigate whether the gains described here have led to improvements in other EmOC signal functions or helped reduce maternal mortality and morbidity at the facility level.

## Conclusion

As Tanzania continues to promote facility births, it is imperative that quality of care during labor and delivery at the facility be improved. AMTSL has the potential to decrease the incidence of PPH, one of the leading causes of maternal death in Tanzania and other LMICs. For AMTSL to be effective, however, it must be performed correctly, following all the recommended steps. The results of this study suggest that this can be achieved by developing and rolling out high-quality, up-to-date, evidence-based guidelines for care of women during labor and delivery. This study also suggests that concentrated quality improvement efforts can contribute to positive changes in PPH prevention practices among health care providers.

This study demonstrated significant improvement in the practice of AMTSL two years after the intervention, with low-level facilities showing greater gains than hospitals. Meaningful change at service delivery points can be brought about by implementation of existing and new guidelines and protocols. Routine measurement of the correct practice of AMTSL among providers of care during labor and delivery can be extremely helpful in monitoring PPH prevention at the facility level.

## Additional files

Additional file 1: QoC Survey LD Checklist: A study tool used to collect observational data during labor and delivery. (PDF 322 kb)

Additional file 2: QoC Survey Inventory Checklist: A checklist used to collect information on health facility readiness for PPH prevention. (PDF 184 kb)

## Abbreviations

AMTSL: Active management of the third stage of labor
CCT: Controlled cord traction
EmOC: Emergency obstetric care
FIGO: International Federation of Gynecology and Obstetricians
ICM: International Confederation of Midwives
LMICs: Low- and middle-income countries
MAISHA: Mothers and Infants Safe, Healthy, Alive
MoHCDGEC: Ministry of Health, Community Development, Gender, Elderly and Children
PPH: Postpartum hemorrhage
QIT: Quality improvement teams
USAID: United States Agency for International Development
WHO: World Health Organization
WIT: Work improvement teams
