# Supplementary material for: Improvement in the active management of the third stage of labor for the prevention of postpartum hemorrhage in Tanzania: a cross-sectional study
Source: BMC Pregnancy Childbirth. 2018 Jun 13;18:223. doi: 10.1186/s12884-018-1873-3 (PMC5998542; doi:10.1186/s12884-018-1873-3)
Supplement: Supplementary file 2 — QoC Survey Inventory Checklist: A checklist used to collect information on health facility readiness for PPH prevention. (PDF 184 kb) [file 12884_2018_1873_MOESM2_ESM.pdf]

MATERNAL AND NEWBORN QUALITY OF CARE FACILITY ASSESSMENT

Essential Inventory

Cover Page	
F1: Facility name	F2: Facility number
F3: Observer number	F4: Today's date<br>(day/month/year)

FIND THE FACILITY DIRECTOR OR IN-CHARGE.

READ TO FACILITY DIRECTOR OR IN-CHARGE.
[insert text of consent script here]

Ask facility director/in-charge Do I have your agreement to proceed?

F5: Was permission received from director/in-charge to participate in study?

☐ Yes, consent is given → go to F6

☐ No, consent is not given → assessment at this facility must END.

F6: Health worker line number<br>(from staff listing)	

Section 1: Infrastructure

READ QUESTIONS ALOUD TO FACILITY DIRECTOR/IN-CHARGE

F100: Does this facility have a working phone or radio system to call outside that is available at all times client services are offered? (clarify that if 24 hour services are offered, this refers to 24 hour availability)	**Code**	**Go to**
Yes, onsite or within 5 mins walk	1	
Yes, within 5 min, not onsite	2	
Only pay phone or personal cell phone	3	
No	4	
F101: Does this facility have a functional ambulance or other vehicle on-site for emergency transportation of clients? IF yes, ask if the vehicle is functioning and if there is fuel available. (Accept reported response.)		
Yes, functioning with fuel	1	
Yes, not functioning or no fuel	2	
No	3	

END OF SECTION 1

Section 2: Labor & Delivery Inventory

ASK TO SPEAK WITH THE HEAD OF LABOR & DELIVERY UNIT (THIS MAY BE DIRECTOR/IN-CHARGE IF NO HEAD OF UNIT)

Question	Yes	No	DK	Go to
f200: Does this facility provide delivery services?	1	0		No→f300
f201: Does this facility provide 24 hour coverage for delivery services?	1	0		No→f203a
f202: Is a person skilled in conducting deliveries present at the facility or on call 24 hours a day, including weekends, to provide delivery care?	**Code**			**Go to**
Yes, present, schedule observed	1			
Yes, present, schedule reported, not seen	2			
Yes, on-call schedule observed	3			
Yes, on-call, schedule reported, not seen	4			
No	5			

READ ALOUD: Now I am going to ask you about medical interventions for management of complications during labor or delivery. For each intervention, please tell me if this is ever provided at this facility, and if yes, if it has been conducted in this facility within the past 3 months.

Question	Yes	No	DK	Go to
F203a: Does this facility ever provide parenteral oxytocic drugs for pregnancy-related hemorrhage	1	0		No→F204a
F203b: In the past 3 months	1	0	8	
F204a: Does this facility ever provide parenteral anticonvulsants for pregnancy-related hypertension	1	0		No→F205a
F204b: In the past 3 months	1	0	8	
F205a: Does this facility ever provide parenteral antibiotics for pregnancy-related infections	1	0		No→F206a
F205b: In the past 3 months	1	0	8	
F206a: Does this facility ever perform manual removal of placenta	1	0		No→F207a
F206b: In the past 3 months	1	0	8	
F207a: Does this facility ever extract retained products of conception	1	0		No→F208a
F207b: In the past 3 months	1	0	8	
F208a: Does this facility ever perform newborn resuscitation	1	0		No→F209a
F208b: In the past 3 months	1	0	8	
F209a: Does this facility ever perform assisted deliveries—that is, use forceps or ventouse (vacuum extractor)	1	0		No→F210a
F209b: In the past 3 months	1	0	8	
F210a: Does this facility ever perform caesarean sections	1	0		No→F211
F210b: In the past 3 months	1	0	8	
F211: Does this facility have a health worker who can perform a caesarean section present in the facility or on call 24 hours a day (including weekends)?	**Code**			**Go to**
Yes, present, schedule observed	1			
Yes, present, schedule reported, not seen	2			
Yes, on-call schedule observed	3			
Yes, on-call, schedule reported, not seen	4			
No	5			
F212: Does this facility have an anesthetist present in the facility or on call 24 hours a day (including weekends)?				
Yes, present, schedule observed	1			
Yes, present, schedule reported, not seen	2			
Yes, on-call schedule observed	3			
Yes, on-call, schedule reported, not seen	4			
No	5			
F213a: Does this facility perform blood transfusions? (IF YES, is there a blood bank or are there transfusion services only)				
Yes, blood bank	1			
Yes, transfusion, no blood bank	2			
No blood transfusion	3			→f214
**Question**	**Yes**	**No**	**DK**	**Go to**
F213b: Has blood transfusion been performed for maternity care by this facility during the past 3 months?	1	0	8	

READ ALOUD: Now I want to ask you about how this facility handles contaminated reusable equipment after completing a delivery. If the unit processes some equipment and sends other equipment elsewhere, indicate the procedure for equipment processed in this service delivery unit. If vaginal deliveries are conducted in a different room than caesarean section deliveries, assess the processing equipment for vaginal deliveries.

F214: After completing a delivery, what procedures do health workers follow for initial handling of contaminated equipment (such as speculums, scalpel handles, etc.) that will be reused another time?	Code	Go to
Disinfectant, then soap & water scrub	1	
Soap & water scrub, then disinfectant soak	2	
Soap & water brush scrub only	3	
Disinfectant soak, not scrubbed	4	
Soap & water, not brush scrubbed	5	
Nothing is done	6	
Other	96	
Don't know	98	
F215: Besides decontaminating and cleaning, what is the final process most commonly used for disinfecting or sterilizing medical equipment (such as surgical instruments) before they are reused? If different methods are used for different types of equipment, indicate the method(s) used for metal equipment such as speculums or forceps.		
Dry-heat sterilization	1	
Autoclaving	2	
Steam sterilization	3	
Boiling	4	
Chemical method	5	
Nothing is done	6	
Other	96	
Don't know	98	

THERE ARE NO MORE QUESTIONS FOR THE HEAD OF LABOR & DELIVERY UNIT/DIRECTOR. EXPLAIN THAT FOR THE NEXT SECTION, YOU WILL NEED TO WALK AROUND AND LOOK AT THE DELIVERY SERVICE AREA. THEY CAN NOW CHOOSE TO ACCOMPANY YOU FOR THE REST OF THE ASSESSMENT OR ATTEND TO OTHER BUSINESS. IF THEY DO NOT ACCOMPANY YOU, ASK IF A HEALTH WORKER INVOLVED IN DELIVERY CARE CAN HELP YOU WITH THE NEXT PART OF THE ASSESSMENT.

ASK TO SEE THE ROOM WHERE NORMAL DELIVERIES ARE CONDUCTED.

F216: Describe the setting of the delivery room	Code	Go to
Private room with visual and auditory privacy	1	
Non-private room with visual and auditory privacy	2	
Visual privacy only	3	
No privacy	4	
F217: Describe the conditions in the delivery room		
Clean	1	
Dirty	2	
Don't Know	3	
F218a: Is there a toilet for client use near the delivery room		
Yes	1	
No	0	→F220
F218b: Is the toilet functioning?		
Yes	1	
No	0	
Don't know	8	

NOTE THE AVAILABILITY AND CONDITION OF THE FOLLOWING SUPPLIES, EQUIPMENT AND MEDICATIONS NEEDED FOR DELIVERY SERVICES. ITEMS MAY BE IN DELIVERY ROOM OR AN ADJACENT ROOM. IF YOU DO NOT SEE AN ITEM, ASK THE HEALTH WORKER HELPING YOU TO SHOW YOU THE ITEM.

SUPPLIES AND EQUIPMENT IN DELIVERY ROOM	Observed	Reported not seen	Not available	Don't know	Go to
F220) Clean and sterile gloves	1	2	3	8	
F221) Sharps container	1	2	3	8	
F222) Already mixed decontaminating solution	1	2	3	8	
F223) Alcohol hand rub	1	2	3	8	
F224) Waste receptacle with lid and plastic liner	1	2	3	8	
F225) Soap for handwashing	1	2	3	8	
F226) Water for handwashing	1	2	3	8	No/DK→F228
**Question**					
F227: How is water being made available for use in the delivery service area today?	**Code**				
Piped	1				
Bucket with tap	2				
Bucket or basin	3				
	**Observed**	**Reported not seen**	**Not available**	**Don't know**	**Go to**

F228) Syringes and Needles	1	2	3	8				
F229) Sterile scissors or blade	1	2	3	8				
F230) Sterile disposable cord ties or clamps	1	2	3	8				
F231) Towel or blanket to wrap baby	1	2	3	8				
F232) Blank partographs or blank maternity booklets with partograph included	1	2	3	8				
	**Availability (a)**					**Functioning (b)**		
	**Observed**	**Reported not seen**	**Not available**	**Don't know**	**Go to**	**Yes**	**No**	**DK**
F235a) Incubator	1	2	3	8	observed→F235b	1	2	8
F236a) Other source of heat for premature infant	1	2	3	8	observed→F236b	1	2	8
F237a) Bag and mask (infant size) for resuscitation	1	2	3	8	observed→F237b	1	2	8
F238a) Penguin sucker for mucus extraction	1	2	3	8	observed→F238b	1	2	8
F239a) Suction apparatus for use with catheter	1	2	3	8	observed→F239b	1	2	8
F240) Resuscitation table for baby	1	2	3	8				
F241a) Forceps	1	2	3	8	observed→F241b	1	2	8
F242a) Ventouse (vacuum extractor - manual or electrical)	1	2	3	8	observed→F242b	1	2	8
F243a) Manual vacuum aspirator (MVA)	1	2	3	8	observed→F243b	1	2	8
F244a) Dilatation and curettage (D&C) kit	1	2	3	8	observed→F244b	1	2	8

IF MEDICATIONS ARE PACKAGED TOGETHER IN A COMBO-PAK, CIRCLE YES FOR EACH INDIVIDUAL MEDICATION IN THE PACK

MEDICATIONS IN DELIVERY ROOM	**Observed ≥1 valid dose**	**Reported not seen**	**Not available**	**Don't know**
F250) Intravenous solutions: either Ringers lactate, D5NS, or NS infusion	1	2	3	8
F251) Injectable ergometrine/ methergine	1	2	3	8
F252) Injectable oxytocin/ syntocin	1	2	3	8
F253) Misoprostol				
F254) Injectable diazepam	1	2	3	8
F255) Injectable magnesium sulfate	1	2	3	8
F256) Injectable phenytoin	1	2	3	8
F257) amoxicillin or injectable ampicillin	1	2	3	8
F258) Injectable gentamicin	1	2	3	8
F259) Zidovudine	1	2	3	8
F260) Lamivudine	1	2	3	8
F261) Nevirapine	1	2	3	8

GUIDELINES MAY BE PRINTED OR HANDMADE.

GUIDELINES/ PROTOCOLS IN DELIVERY ROOM	**Observed**	**Reported not seen**	**Not available**	**Don't know**
F265: Guidelines for care/managing normal labor and birth	1	2	3	8
F266: Guidelines for emergency obstetric care	1	2	3	8
F267: Guidelines for PMTCT	1	2	3	8

ASK TO SEE THE ROOM WHERE CAESAREAN SECTIONS / SURGERIES ARE PERFORMED. IF THERE IS NO ROOM FOR SURGERIES, LOOK IN THE DELIVERY ROOM. CHECK IF THE FOLLOWING EQUIPMENT, SUPPLIES AND MEDICATIONS ARE AVAILABLE IN THE ROOM OR IN AN ADJACENT ROOM.

	**Availability (a)**					**Functioning (b)**		
EQUIPMENT IN SURGICAL SERVICE AREA	**Observed**	**Reported not seen**	**Not available**	**Don't know**	**Go to**	**Yes**	**No**	**DK**
F270a) Operating Table	1	2	3	8	observed→F270b	1	2	8
F271a) Operating light	1	2	3	8	observed→F271b	1	2	8
F272a) Anesthesia giving set	1	2	3	8	observed→F272b	1	2	8
F273) Scrub area adjacent to or in the operating room	1	2	3	8				
F274) Tray, drum, or package with sterilized instruments ready for use	1	2	3	8				

MEDICATIONS IN SURGICAL SERVICE AREA	**Observed ≥1**	**Reported not seen**	**Not available**	**Don't know**

	**valid dose**							
F275) Halothane	1	2	3	8				
F276) Ketamine	1	2	3	8				

ASK THE HEALTH WORKER TO SHOW YOU WHERE DELIVERY EQUIPMENT IS STERILIZED.

	**Availability (a)**					**Functioning (b)**		
EQUIPMENT USED FOR STERILIZATION	**Observed**	**Reported not seen**	**Not available**	**Don't know**	**Go to**	**Yes**	**No**	**DK**
F280a) Electric autoclave (Pressure and Wet Heat)	1	2	3	8	observed→F280b	1	2	8
F281a) Non-electric autoclave (Pressure and Wet Heat)	1	2	3	8	observed→F281b	1	2	8
F282a) Electric dry heat sterilizer	1	2	3	8	observed→F282b	1	2	8
F283a) Electric boiler or steamer (no pressure)	1	2	3	8	observed→F283b	1	2	8
F284) Non-electric pot with cover (for steam/boil)	1	2	3	8				
F285a) Heat source for non-electric equipment	1	2	3	8	observed→F285b	1	2	8
F286a) Automatic Timer (May be on equipment)	1	2	3	8	observed→F286b	1	2	8
F287) TST Indicator strips or other item that indicates when sterilization is complete.	1	2	3	8				
F288) chlorine-based or glutaraldehyde solution (for chemical method)	1	2	3	8				
F289) Written protocols or guidelines for sterilization or disinfection	1	2	3	8				

END OF SECTION 2

Section 3: Antenatal Care Inventory

ASK TO SPEAK WITH THE HEAD OF ANTENATAL CARE UNIT (THIS MAY BE DIRECTOR/IN-CHARGE IF NO HEAD OF UNIT)

**Question**	**Yes**	**No**			**Go to**
F300: Does this facility offer routine antenatal services?	1	0			
F301: Does this facility offer referral antenatal services?	1	0			F300 is no and F301 is no→end section
F302: Does this facility have a system whereby measurements or procedures for ANC clients are routinely carried out before the consultation?	1	0			No→F308

ASK TO SEE THE PLACE WHERE ANTENATAL CLIENTS ARE SEEN BEFORE THEY HAVE THEIR MEDICAL CONSULTATION AND INDICATE WHICH OF THE FOLLOWING ACTIVITIES ARE ROUTINELY CARRIED OUT THERE. OBSERVE IF THE BELOW ACTIVITIES ARE BEING CONDUCTED ROUTINELY. IF NOT SEEN ASK:

Is [read activity you do not see] routinely conducted for all antenatal care clients?	**Observed**	**Reported not seen**	**Not available**	**Don't know**	**Go to**
F303: Weighing clients	1	2	3	8	
F304: Taking blood pressure	1	2	3	8	
F305: Urine test for protein	1	2	3	8	
F306: Blood test for anemia	1	2	3	8	
F307: Conducting group health education sessions	1	2	3	8	

Which of the following activities are performed as part of routine services, that is, each client has this test at least once.	**Yes**	**No**	**DK**	**Go to**
F308: Blood test for anemia	1	0	8	
F309: Blood test for syphilis	1	0	8	
F310: Blood group	1	0	8	
F311: Test for Rh factor	1	0	8	
F312: Urine test for protein	1	0	8	
F313: Urine test for glucose	1	0	8	
Which of the following types of treatment and services are routinely offered to antenatal clients?				
F314: SP for Intermittent Preventive Therapy for malaria	1	0	8	
F315: Counseling about family planning	1	0	8	
F316: Counseling about HIV/AIDS	1	0	8	
F317: Testing for HIV/AIDS	1	0	8	

F318: Is tetanus toxoid vaccination available all days antenatal care services are offered?	**Code**	**Go to**
Yes	1	
Not all days	2	
Never offered	3	
F319: How many days each week are tetanus toxoid vaccinations offered at this facility? (If never offered, enter 0, don't know enter 8)		
F320: Is tetanus toxoid immunization available today?		
Yes	1	
No	0	

THERE ARE NO MORE QUESTIONS FOR THE HEAD OF ANTENATAL CARE UNIT/DIRECTOR. EXPLAIN THAT FOR THE NEXT SECTION, YOU WILL NEED TO WALK AROUND AND LOOK AT THE ANTENATAL CARE EXAMINATION AREA. THEY CAN NOW CHOOSE TO ACCOMPANY YOU FOR THE REST OF THE ASSESSMENT OR ATTEND TO OTHER BUSINESS. IF THEY DO NOT ACCOMPANY YOU, ASK IF A HEALTH WORKER INVOLVED IN ANTENATAL CARE CAN HELP YOU WITH THE NEXT PART OF THE ASSESSMENT.

ASK TO SEE THE ROOM WHERE EXAMINATIONS FOR ANTENATAL CLIENTS ARE CONDUCTED.

F325: Describe the setting of the ANC examination room	**Code**	**Go to**
Private room with visual and auditory privacy	1	
Non-private room with visual and auditory privacy	2	
Visual privacy only	3	
No privacy	4	

F326: Describe the conditions in the ANC examination room								
Clean	1							
Dirty	2							
Don't Know	3							
F327a: Is there a toilet for client use near the ANC service delivery area	**Code**				**Go to**			
Yes	1							
No	0				→F328			
F327b: Is the toilet functioning?								
Yes	1							
No	0							
Don't know	8							

Note the availability and condition of the following supplies, equipment and medications needed for ANC services. Items may be in the room where ANC examinations take place or an adjacent room. If you do not see an item, ask the health worker helping you to show you the item.

SUPPLIES AND EQUIPMENT IN ANC EXAMINATION ROOM	**Observed**	**Reported not seen**	**Not available**	**Don't know**	**Go to**
F328) Clean and sterile gloves	1	2	3	8	
F329) Sharps container	1	2	3	8	
F330) Already mixed decontaminating solution	1	2	3	8	
F331) Alcohol hand rub	1	2	3	8	
F332) Waste receptacle with lid and plastic liner	1	2	3	8	
F333) Soap for handwashing	1	2	3	8	
F334) Water for handwashing	1	2	3	8	No/DK→F338a

**Question**	**Code**
F335: How is water being made available for use in the delivery service area today?	
Piped	1
Bucket with tap	2
Bucket or basin	3

Equipment may be in examination room, an adjacent room, or room where measure is taken.

EQUIPMENT AND TESTING SUPPLIES	**Observed**	**Reported not seen**	**Not available**	**Don't know**	**Go to**	**Yes**	**No**	**DK**
F338a) Blood pressure apparatus	1	2	3	8	observed→F338b	1	2	8
F339a) Stethoscope	1	2	3	8	observed→F339b	1	2	8
F340a) Fetal stethoscope (Fetoscope)	1	2	3	8	observed→F340b	1	2	8
F341a) Adult weighing scale	1	2	3	8	observed→F341b	1	2	8
F342) Urine test strip for protein	1	2	3	8				
F343) RPR kit / Syphilis SD Bioline	1	2	3	8				
F344) HIV rapid test / HIV Determine and Unigold	1	2	3	8				

MEDICATIONS/ VACCINE	**Observed**	**Reported not seen**	**Not available**	**Don't know**	**Go to**
F345) Iron and/or folic acid	1	2	3	8	
F346) Tetanus toxoid vaccine	1	2	3	8	
F347) SP (Fansidar)	1	2	3	8	
F348) Mebendazole/Albendazole	1	2	3	8	

If medications are packaged together in a combo-pak, circle yes for each individual medication in the pack

	**Availability (a)**			
ARV MEDICATIONS	**Observed ≥1 valid dose**	**Reported not seen**	**Not available**	**Don't know**
F349) Zidovudine	1	2	3	8
F350) Lamivudine	1	2	3	8
F351) Nevirapine	1	2	3	8

End of Section 3
